# Repetitive nonreentrant ventriculoatrial synchrony inducing atrial fibrillation in setting of dofetilide

**DOI:** 10.1016/j.hrcr.2022.01.015

**Published:** 2022-02-04

**Authors:** Leonid Garber, Eric Shulman, Alexander Kushnir, Tajinderpal Saraon, David S. Park, Larry A. Chinitz

**Affiliations:** organization Leon H. Charney Division of Cardiology, Cardiac Electrophysiology, NYU Langone Health, New York University Grossman School of Medicine, New York, New York

**Keywords:** Pacemaker, Repetitive nonreentrant ventriculoatrial synchrony, Atrial fibrillation, Dofetilide, Pacemaker programming

## Introduction

Repetitive nonreentrant ventriculoatrial synchrony (RNRVAS) is a type of pacemaker-mediated arrhythmia in patients with dual-chamber devices programmed in an atrial tracking mode (DDD or DDDR). It is important to recognize RNRVAS and pacemaker-mediated tachycardia (PMT), as both of these ventriculoatrial (VA) synchrony arrhythmias may cause symptoms and induce further atrial tachyarrhythmias, thus highlighting the need for recognition and appropriate device programming to prevent their occurrence. We describe a case of RNRVAS-induced atrial fibrillation (AF) in a patient on dofetilide.

**Key Teaching Points**

• Repetitive nonreentrant ventriculoatrial synchrony (RNRVAS) may occur with dual-chamber devices programmed in atrial tracking mode. There is repetitive retrograde ventriculoatrial conduction where retrograde atrial activation falls within the postventricular atrial refractory period, with subsequent atrial pacing during the absolute refractory period, leading to functional atrial noncapture and triggering ventricular pacing at the programmed paced atrioventricular (AV) delay.
• RNRVAS may lead to atrial fibrillation initiation by pacing the atrium in the vulnerable period. Decreasing the lower rate limit or the paced AV delay will increase the V-to-AP interval and reduce the chance of functional atrial noncapture, thereby lowering the risk of RNRVAS.
• Dofetilide increases the refractory period of the atria and may theoretically contribute to the development of RNRVAS by increasing the chance of functional atrial noncapture. Inhibition of hERG/IKr channels may increase the likelihood of an unfortunately timed paced depolarization landing during the vulnerable period of the atrium and inducing fibrillation.

## Case report

A patient with an ischemic cardiomyopathy (left ventricle ejection fraction of 20%) and a dual-chamber implantable cardiac defibrillator (ICD) (ENERGEN ICD E143; Boston Scientific, Natick, MA) was admitted to the hospital with acute decompensated heart failure. He has a history of coronary artery disease with prior myocardial infarction and coronary arterial bypass grafting, as well as paroxysmal AF. Owing to syncope, sinus node dysfunction, and a reduced ejection fraction from ischemic cardiomyopathy, he underwent implantation of a dual-chamber ICD. Several years later, he was started on treatment with dofetilide for symptomatic episodes of atrial fibrillation. Owing to persistent symptomatic episodes despite dofetilide use, he underwent radiofrequency ablation with pulmonary vein isolation and posterior wall isolation. Three months after ablation, dofetilide was restarted by the patient’s cardiologist for an episode of atrial tachycardia (AT) and the lower rate limit (LRL) was increased from 60 to 70.

**Figure 1:**
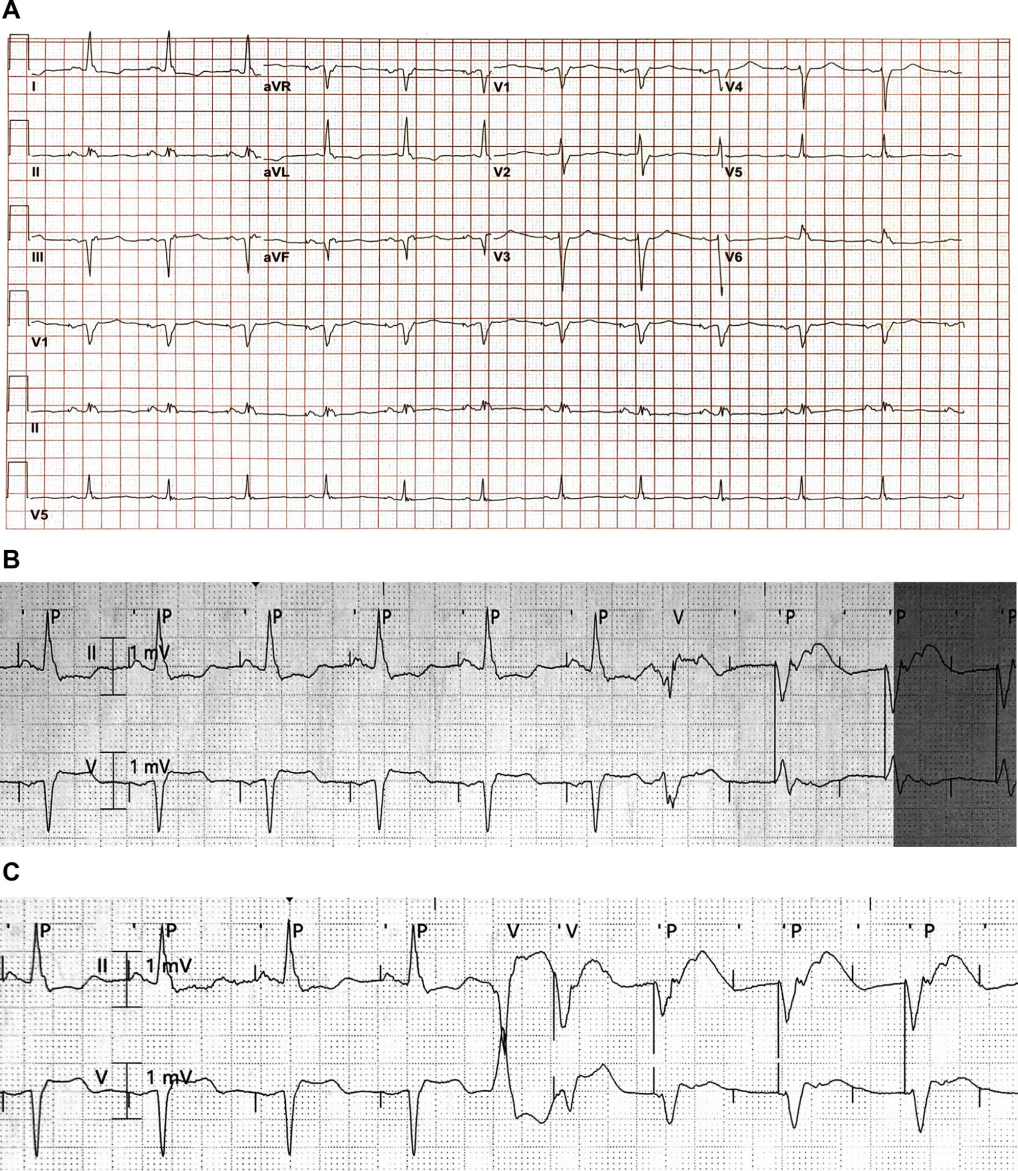
**A:** Baseline electrocardiogram: atrial paced rhythm at 70 beats per minute, inferior infarction, and a left bundle branch block morphology with QRS widening and associated repolarization abnormalities. **B:** Telemetry recording with premature ventricular contraction (PVC) with retrograde atrial conduction during the postventricular atrial refraction period initiates a cycle of atrial paced functional noncapture and ventricular pacing with a prolonged atrioventricular delay of 350 ms, consistent with RNRVAS**. C:** Telemetry recording of a PVC with pseudo-pseudo fusion that was not sensed because it fell in the postatrial ventricular blanking period, followed by ventricular pacing with retrograde atrial conduction outside of the PVARP that initiates the cycle of RNRVAS.

During his admission for acute decompensated heart failure, initial 12-lead electrocardiogram revealed an atrial paced rhythm at 70 beats per minute, inferior infarction, and a left bundle branch block morphology with QRS widening and associated repolarization abnormalities (Figure 1A). Baseline device programming was DDD 70–100, paced atrioventricular (AV) delay 280–350 ms, sensed AV delay 280–350 ms, postventricular atrial refractory period (PVARP) 300–320 ms.

**Figure 2:**
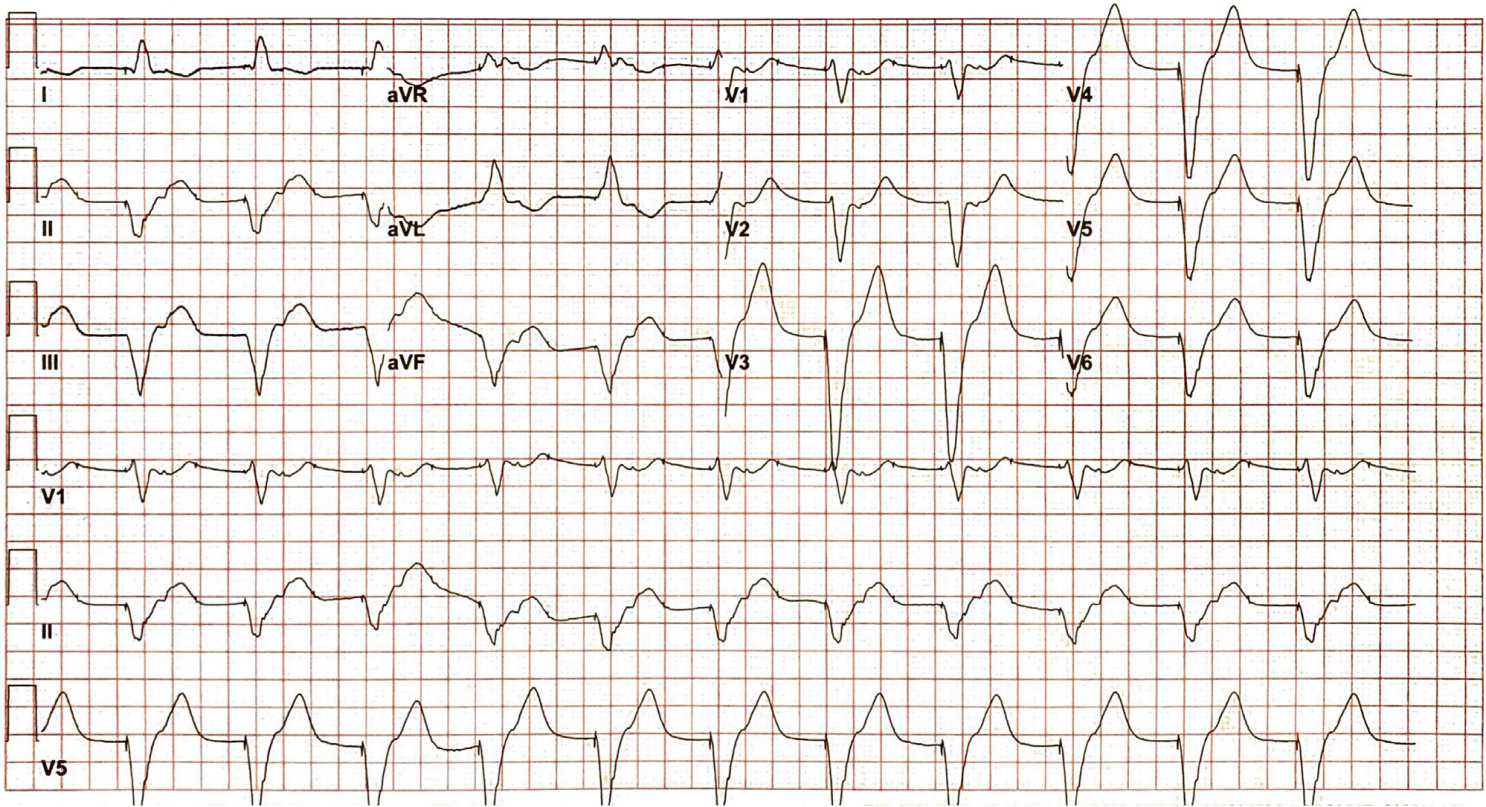
Repetitive nonreentrant ventriculoatrial synchrony on 12-lead electrocardiogram.

Upon review of telemetry during the admission, several episodes of a ventricular paced rhythm were noted. In Figure 1B, a premature ventricular contraction (PVC) with retrograde atrial conduction during the PVARP initiates a cycle of atrial paced (AP) functional noncapture and ventricular pacing with a prolonged AV delay of 350 ms. In Figure 1C, a PVC with pseudo-pseudo fusion that was not sensed because it fell in the postatrial ventricular blanking period was followed by ventricular pacing with retrograde atrial conduction outside of the PVARP, initiating the cycle of RNRVAS. A 12-lead electrocardiogram capturing this rhythm is depicted in Figure 2.

**Figure 3:**
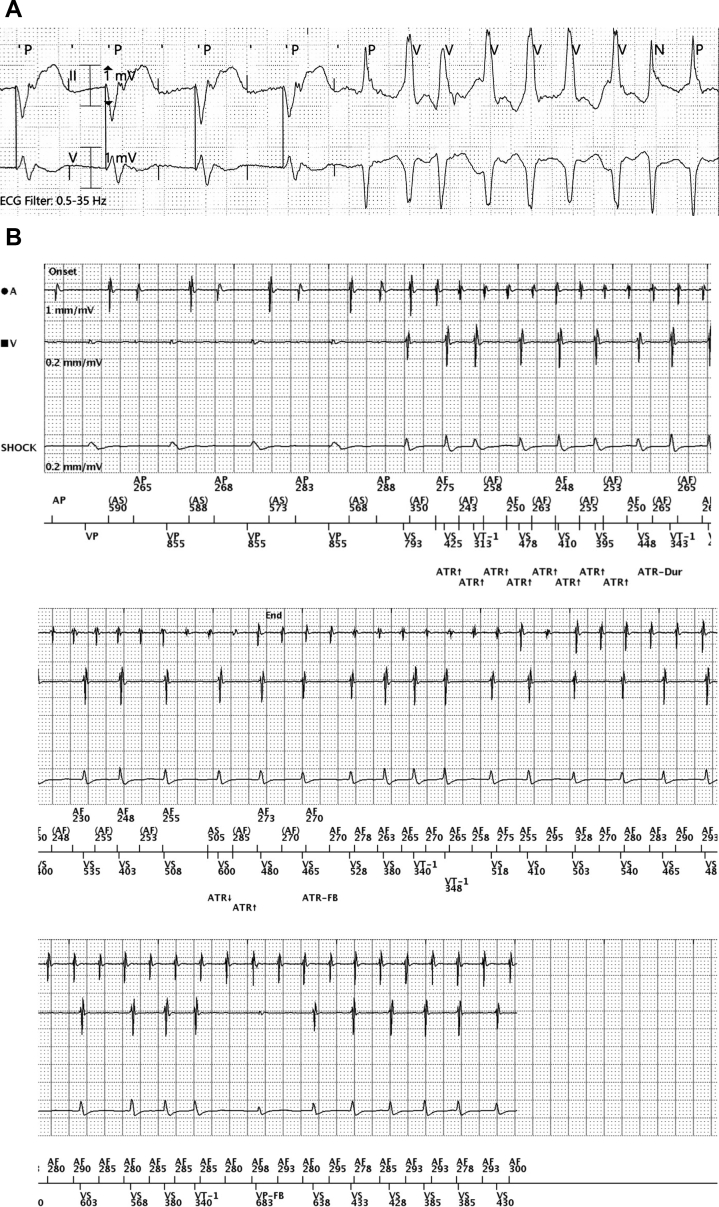
**A:** Telemetry recording of repetitive nonreentrant ventriculoatrial synchrony initiating atrial fibrillation (AF) with atrial pacing. **B:** Electrogram recording of episode during which atrial pacing captures the atrial myocardium and triggers AF.

During the course of hospitalization, he experienced symptomatic recurrence of AF. Review of the telemetry recording at the onset of AF shows atrial pacing during the vulnerable period preceding the onset of AF (Figure 3A). Interrogation of his dual-chamber ICD corroborates the findings of multiple episodes of Atrial Tachy Response (ATR) mode switch from AF with rapid ventricular response, all initiated by atrial pacing in the vulnerable period during episodes of RNRVAS (Figure 3B). Since restarting dofetilide and increasing the LRL from 60 to 70, 10 atrial high rate episodes (AHRE) correlating to atrial fibrillation were detected on device interrogation over the course of 2 months.

The device was reprogrammed from DDD 70–100 to an LRL of 60 and dofetilide was stopped. Upon follow-up of 10 months’ duration, he reported no further symptomatic episodes of AF and device interrogation showed no recurrences of ATR mode switch episodes.

## Discussion

RNRVAS and PMT (also referred to as endless loop tachycardia) are 2 types of VA arrhythmias that occur with dual-chamber devices programmed in atrial tracking mode. In the less commonly encountered RNRVAS, there is repetitive retrograde VA conduction where the retrograde atrial activation falls within the PVARP with subsequent atrial pacing during the absolute refractory period, leading to functional atrial noncapture. Atrial pacing without capture still triggers ventricular pacing at the programmed paced AV delay and continues the cycle of RNRVAS.^1^

RNRVAS has been independently associated with occurrence of AT and AF. A recent study from the RATE registry has shown that the induction of AF by atrial pacing events occurred in 62% of analyzed episodes where competitive atrial pacing preceded AF initiation.^2^ The proposed mechanism responsible for this is related to a pacing stimulus delivered during the relative refractory period of the atrium, also known as the vulnerable period.^2^^,^^3^

Only older Abbott devices capture RNRVAS episodes because they trigger spurious detection of AHRE. These events were captured in Abbott devices by detecting both the retrograde conducted A falling in the PVARP and the noncaptured A pacing, both of which counted toward the mode switch trigger. On Abbott’s newer devices (Assurity, Endurity, and Gallant), AP events do not count toward the atrial counter for AHRE. In other devices, assessment of initiation of AHRE in the setting of RNRVAS triggering AF may be fortuitously detected. Otherwise, there are no current algorithms specifically programmed to capture RNRVAS episodes.

Reprogramming of intracardiac devices to reduce the risk of RNRVAS and its associated adverse events is possible via several adjustments to the pacing parameters. Decreasing the LRL or removing rate-responsive pacing will increase the V-to-AP interval and reduce the chance of functional atrial noncapture and the risk of atrial pacing during the vulnerable period. Decreasing the paced AV delay will similarly extend the V-to-AP duration, but may increase the overall percentage of RV pacing.^4^ Shortening the PVARP is generally not recommended, as it may increase the likelihood of PMT.^5^ In Medtronic devices, Non-Competitive Atrial Pacing (NCAP) can be programmed, which will automatically trigger a 300 ms NCAP period after an atrial sensed event within the PVARP, during which no atrial pacing will occur. The NCAP period prevents pacing within the atrium’s relative refractory period, thereby preventing a potential trigger of AT and AF initiation. Similarly on Abbott Gallant devices, an algorithm called PAC Response reduces the frequency of RNRVAS by delaying atrial pacing after an atrial sensed event in the PVARP with a programmable interval called the PAC Response Interval. The use of pacing modes that allow AAI pacing with a DDD or VVI backup, such as MVP (Medtronic) or RHYTHMIQ (Boston Scientific) modes, will also reduce the frequency of RNRVAS.^4^^,^^5^

Although not previously described, the use of dofetilide or other antiarrhythmics that strongly increase the refractory period of the atria may theoretically contribute to the development of RNRVAS by increasing the chance of functional atrial noncapture. Moreover, one may speculate that by extending the action potential with inhibition of hERG/IKr channels, dofetilide may potentially increase the likelihood of an unfortunately timed paced depolarization landing during the vulnerable period of the atrium and inducing fibrillation, even at lower atrial pacing rates with prolonged VA intervals. Patients on dofetilide and other QTc-prolonging agents may also be programmed at a higher LRL to prevent the occurrence of bradycardia-related torsades de pointes, as in our patient, who was initially programmed at an LRL of 70. These factors may all explain how dofetilide can increase the risk of RNRVAS and development of competitive atrial pacing–induced AF. Although the patient in our case report had no further AF recurrence after stopping dofetilide during follow-up, simultaneous device reprogramming to an LRL of 60 from 70 and medical therapy optimization by an advanced heart failure specialist after his hospitalization may have also contributed to cessation of AF recurrences.

## Conclusion

RNRVAS is a relatively uncommon arrhythmia in patients with dual-chamber devices in atrial tracking mode and may be missed by routine device interrogation. Reprogramming is important to prevent symptoms and triggering of atrial tachyarrhythmias and AF. Patients on dofetilide may be at increased risk of developing RNRVAS.
